# Erchen decoction combined with Sanziyangqin decoction for chronic obstructive pulmonary disease: A protocol for systematic review and meta-analysis

**DOI:** 10.1097/MD.0000000000022315

**Published:** 2020-10-02

**Authors:** Lu Deng, Xinyue Zhang, Yan Dong, Lizhen Wang, Keling Chen, Meiling Zheng, Zidanqing Yang, Hui Tang, Wenhao Liao, Qunfeng Shi

**Affiliations:** a Hospital of Chengdu University of Traditional Chinese Medicine; b College of Health Preservation and Rehabilitation, Chengdu University of Traditional Chinese Medicine, Chengdu, Sichuan Province, China.

**Keywords:** chronic obstructive pulmonary disease, erchen decoction, protocol, sanziyangqin decoction, systematic review and meta-analysis

## Abstract

**Introduction:**

chronic obstructive pulmonary disease (COPD) is 1 of the leading causes of morbidity and mortality worldwide; its economic and social burdens are substantial and increasing. Recent years, an increasing number of study has shown the promising advantage of Erchen decoction (ECD) combined with sanziyangqin decoction (SZYQD) in treating COPD. However, due to the lack of evidence, there is no specific method or suggestion, so it is necessary to provide a protocol for a systematic review on ECD combined with SZYQD for COPD and provide effective evidence for further clinical use.

**Methods and analysis:**

We will conduct a Computerized literature searches in the following databases: PubMed, MEDLINE, EMBASE, Cochrane Library, China national information network, China biomedical literature database (CBM), Chinese Scientific Journals Database and wanfang database, from their inception to June 2020, without restrictions of language. Study selection, data collection, and evaluation of the quality of evidence will be performed by 2 researchers independently, risk of bias of the meta-analysis will be evaluated based on the Cochrane Handbook for Systematic Reviews of Interventions. All data analysis will be conducted by data statistics software Review Manager V.5.3. and Stata V.12.0.

**Results:**

This study will systematically evaluate the effectiveness and safety of ECD combined with SZYQD for COPD. The results will be published in a peer-reviewed journal.

**Conclusion:**

This study will provide evidence from the current published RCTs of whether ERD combined with SZYQD is an effective and safe intervention for COPD.

**Ethics and dissemination:**

This study is a systematic review, the outcomes are based on the published evidence. In this study, no individual data from participants will be involved, so ethics approval is not required.

**Open Science Framework(OSF)registration number:**

August 19, 2020; osf.io/zxm24.

## 1 Introduction

### 1.1 Description of the condition

Chronic obstructive pulmonary disease (COPD) is a common disease characterized by persistent airflow limitation that can be prevented and treated. COPD has become 1 of the major chronic diseases threatening human health.^[1]^ According to the World Health Organization (WHO), there were 251 million COPD patients worldwide in 2016, and an estimated 3.17 million people died of COPD in 2015, making COPD the third leading cause of death worldwide. The Global Burden of Disease Study (GBD) estimates that annual disability rates in COPD exceed 5 million, ranking third in the list of causes of disability-adjusted life years lost.^[2]^

In recent years, more and more studies showed the curative effect of Traditional Chinese medicine (TCM) treatment for COPD.[^3^,^4^] Chinese herbs can improve the patients’ clinical symptoms, physical signs, the quality of survival, and the patients’ lung function, also, reduce the number of Acute exacerbation, and the morbidity and mortality through the anti-inflammatory, antitussive expectorant, adjust oxidation - anti-oxidation mechanism, and regulating immune system.

### 1.2 Description of the intervention

TCM has been used more than thousands of years for respiratory disease in Asian countries, especially in China. Erchen decoction (ECD) combined with sanziyangqin decoction (SZYQD) is composed of 7 kinds of TCM herbs (RHIZOMA PINELLIAE EXOCARPIUM CITRI RUBRUM PORIA GlYCYRRHIZA URALENSES SEMEN RAPHANI SEMEN SINAPIS FRUCTUS PERILLAE). ECD combined with SZYQD has been often used in treating COPD in clinical practice in China.

### 1.3 How the intervention might work

According to the TCM theory, “Phlegm retention” is the main pathogenic factor of COPD and also the important link that leads to the aggravation of disease. Thus, ECD combined with SZYQD can be used to treat COPD by Invigorating the spleen and reduce dampness and phlegm. Modern pharmacological research has found that ECD combined with SZYQD can improve the state of airway mucous hypersecretion of COPD by regulating inflammatory markers such as TNF-α and neutrophils. And thus improve the clinical symptoms and indicators caused by airway mucous hypersecretion.

### 1.4 Why it is important to this review

ECD combined with SZYQD is a common treatment in COPD in TCM, more and more researches have been carried out in the treatment of COPD, and some suggested that ECD combined with SZYQD is more effective than conventional treatment alone.[^5^,^6^] However, currently no critical evidence for systematic evaluation or meta-analysis of the potential advantages and harms of ECD combined with SZYQD in the treatment of COPD. We aim to assess the available evidence of ECD combined with SZYQD for COPD according to randomized controlled trials (RCTs).

### 1.5 Objectives

To our knowledge, there is no systematic review of efficacy and safety of ECD combined with SZYQD in the treatment of COPD. Therefore, we hope to providing a reference for the treatment of COPD in clinical use.

## 2 Methods

### 2.1 Study registration

The protocol of the systematic review has been registered. Registration:OSF Preregisration.OSF Preregisration. August 19, 2020; osf.io/zxm24. We will complete this protocol according to the Preferred Reporting Items for Systematic reviews and Meta-Analysis Protocols statement guidelines.^[7]^ And the important protocol amendments will be documented in the full review.

### 2.2 Criteria for considering studies for this review

We will strictly screen studies according to the PICOS strategy.

#### 2.2.1 Type of included studies

Only RCTs (except Quasi-RCTs and cluster RCTs) regarding ECD combined with SZYQD for the treatment of COPD will be Involved. Animal mechanism studies and non-randomized clinical trials will be excluded. The language and time of publication will not be restricted.

#### 2.2.2 Type of Participants

We will include RCTs of participants with COPD as per the criteria for diagnosis based on the Global Initiative for Chronic Obstructive Lung Disease,^[1]^ or guideline of Chinese Medical Association Respiratory Diseases Society for COPD.^[8]^ Regardless of age, gender, region, and other factors. Patients who suffered from other respiratory system diseases will be excluded.

#### 2.2.3 Type of Interventions and controls

We will only include studies which interventions included ECD combined with SZYQD for treatment alone or combined with other conventional treatment. The control group received only conventional treatment. The choice of conventional treatment for each RCT do not need to be completely consistent. There will be no limitation on the duration, and dosage of ECD combined with SZYQD.

#### 2.2.4 Type of outcome measures

Primary outcomes:

1) Lung function, including the FEV1, FVC, FEV1/predicted value (FEV1%), and FEV1/FVC. Lung function examination is a good objective index of repeat-ability in determining airflow limitation, and is of great significance in the diagnosis, severity evaluation, disease progression, prognosis and treatment response of COPD Flow limitation is determined by FEV1 and FEV1/FVC reductions. FEV1/FVC is a sensitive indicator of copd and can detect airflow limitation to guide chronic obstructive lung disease classification. FEV1% is a good indicator for evaluating moderate and severe airflow limitation. Due to its small variability and easy operation, FEV1% is regarded as a basic item for pulmonary function examination of COPD.;
2) Frequency and duration of acute exacerbation of COPD during follow-up after study entry.

Secondary outcomes:

(1) COPD assessment test (CAT). Compared with The St. George's Breathing Questionnaire, CAT has very similar assessment ability, but it takes much less time, also, it is easy to calculate, can be completed independently by the patient, and is reproducible, authentic and sensitive to the patient. Therefore, it is more suitable for routine clinical use;
(2) Clinical effective rates;
(3) Scores of TCM syndrome.

### 2.3 Search methods

#### 2.3.1 Search resources

This review will include the following electronic databases from their inception to 15 August 2020: PubMed, MEDLINE, EMBASE, Cochrane Library, China national information network, China biomedical literature database (CBM), Chinese Scientific Journals Database and wanfang database.

#### 2.3.2 Search strategies

The following terminology will be used to search the databases above: (Erchen decoction ADN Sanziyangqin decoction) combined with (COPD) combined with (RCT OR RCTs). Search strategies in Pubmed is shown in Table 1.

**Table 1:**
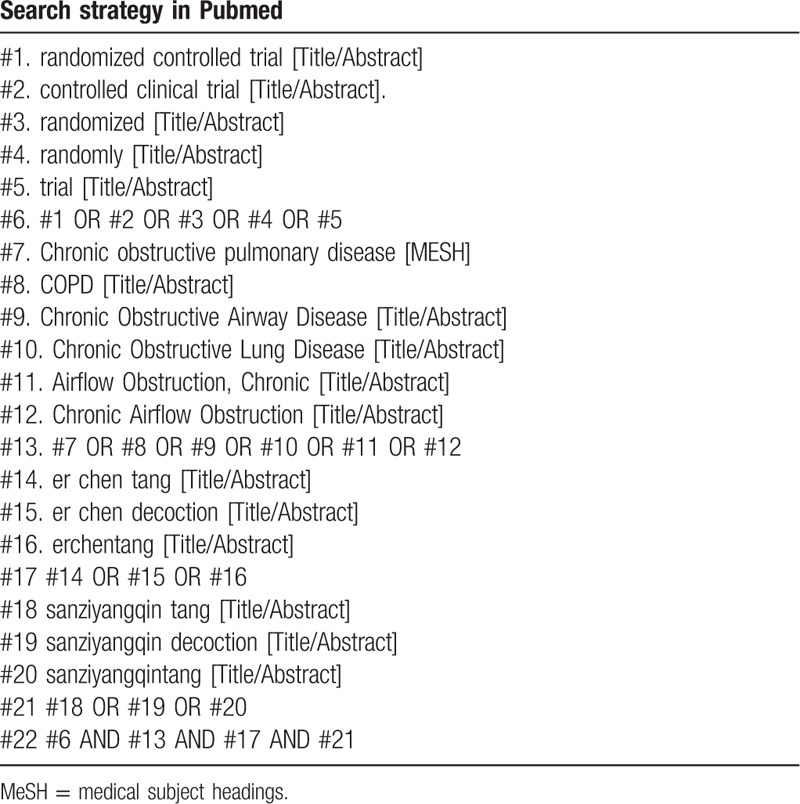
The search strategy. Table 1. Search strategy in Pubmed.

#### 2.3.3 Searching other resources

Google Scholar and Baidu Academic will be involved to search relevant literature. In addition, in order to ensure the comprehensiveness of the search. we will manually retrieve references for all eligible studies.

### 2.4 Studies selection

**Figure 1:**
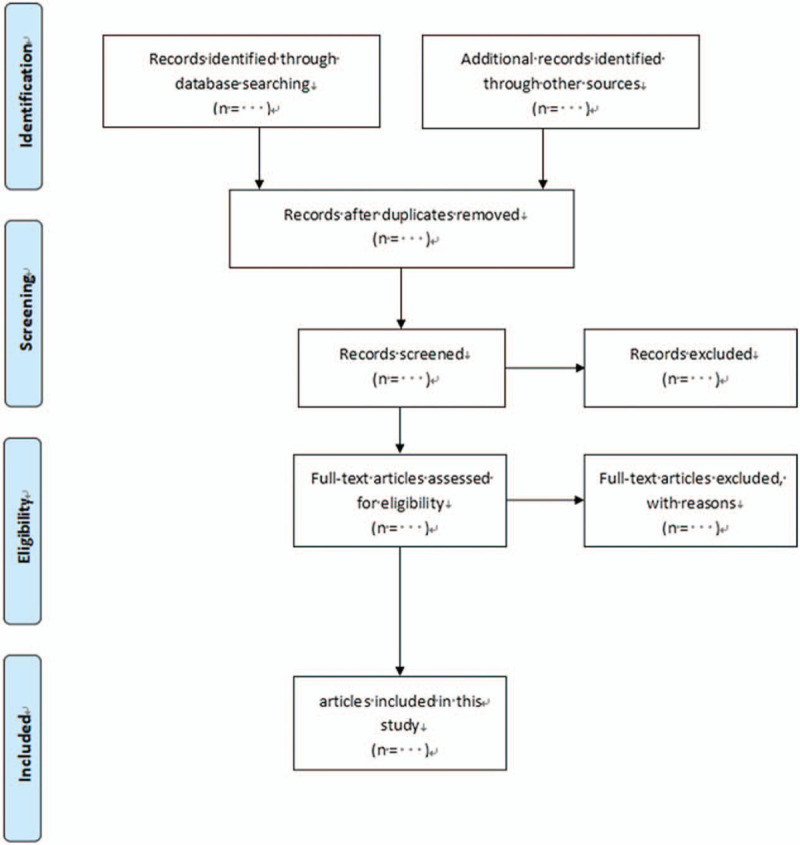
The research flowchart. This figure shows the identification, screening, eligibility and included when we searching articles.

The titles and abstracts of all retrieved articles will be screened first, full articles of potential will be obtained according to the inclusion criteria second. This process will be conducted by 2 authors independently (LD and XY-Z). Disagreements will be resolved by discussion and a third reviewer's introduction (ML-Z). The detailed flow of literature selection is shown in Figure 1.

### 2.5 Data extraction

Two authors (LD and XY-Z) will extract data independently. Disagreements between authors will be resolved by discussing and the consulting of the third author (ML-Z). Study data will be extracted using extraction forms designed specifically for this review

(1) general information, including trial name and registration information;
(2) trial characteristic, including trial design, location, setting, and inclusion/exclusion criteria;
(3) characteristic of participants, including age, sex, race/ethnicity, severity of COPD;
(4) details of interventions, including dose, duration of treatment, co-interventions (conventional treatment);
(5) details of comparison interventions;
(6) outcomes including lung function, frequency and duration of acute exacerbation, CAT, clinical effective rates, TCM syndrome, and adverse events.;
(7) adverse events.

### 2.6 Assessment of risk of bias

All the included studies will be evaluated based on the guidelines of Cochrane Handbook for Systematic Reviews of Interventions.^[9]^ The quality of each trial will categorized into ‘low’, ‘unclear’, or ‘high’ risk of bias according to the following items: adequacy of generation of the allocation sequence, allocation concealment, blinding of participants and personal, blinding of outcome assessors, incomplete outcome data, selected reporting the results and other sources of bias (such as comparable baseline characteristic, inclusion and exclusion criteria).

### 2.7 Assessment of publication biases

Publication biases and small-study effects will be detected by funnel plot and Egger test if there are 10 more studies included in this Meta- analysis. For Egger test, *P* value of < .10 was considered to indicate the exist of publication biases and small study effects.

### 2.8 Dealing with missing data

We will contact the first or corresponding author to get missing information from their trials and use the available data for data synthesis. If the necessary data are unobtainable, the impact of missing data will be discussed.

### 2.9 Assessment of heterogeneity

We will assess the Statistical heterogeneity by *X* ^2^ and *I* ^2^ statistical tests. Where *P* value ≥.1 and *I* ^2^ ≤50%, there is no obvious statistical heterogeneity among the studies. On the contrary, where *P* value <.1or *I* ^2^ > 50% indicates a considerable heterogeneity. Meta-analysis will be performed when the statistical heterogeneity is acceptable (*P* value ≥.1and *I* ^2^ ≤50%), otherwise, as large heterogeneity, subgroup or sensitivity analysis will be conducted.

### 2.10 Data synthesis

RevMan software (Version 5.3) will be used to perform data synthesis.Binary outcomes will be summarized using risk ratio with 95% confidence interval for relative effect. Continuous outcomes will be summarized by using weighted mean difference with 95% confidence interval. The fixed effects model will be performed for meta-analysis when there is small homogeneity (*I* ^2^ < 50%). If not, the random effects model will be conducted. If there is a significant heterogeneity exist among studies, we will perform subgroup analysis, sensitivity analysis or descriptive analysis

### 2.11 Subgroup analysis

Subgroup analysis will be conducted based on different factors, including, participants, dose of medication, duration of treatment, severity of COPD.

### 2.12 Sensitivity analysis.

Sensitivity analysis will be conducted if there are sufficient data available.

### 2.13 Evidence assessed

The quality of evidence for this study will be assessed by “Grades of Recommendations Assessment, Development and Evaluation”(GRADE) standard established by the World Health Organization and international organizations.^[10]^ To achieve transparency and simplification, the quality of evidence is divided into 4 levels in GRADE system: high, medium, low and very low. We will employ GRADE profiler 3.2 for analysis.

### 2.14 Patient and public involvement

Patient and public were not involved in this study.

### 2.15 Ethics and dissemination

Ethical approval will not be required for this systematic review. The results of this review will be disseminated by being published in a peer-reviewed journal.

## 3 Discussion

COPD is a progressive and chronic lung disease which generally associated with symptoms such as cough, sputum, production, and dyspnea.^[11]^ ERD, consisting of RHIZOMA PINELLIAE EXOCARPIUM CITRI RUBRUM PORIA GlYCYRRHIZA URALENSES, is a famous prescription in TCM and has the effect of eliminating phlegm. Studies[^12^,^13^] have shown that both RHIZOMA PINELLIAE (Ban Xia) and PORIA (Fu Ling) have sedative effects, which may improve the quality of sleep in COPD patients. Moreover, RHIZOMA PINELLIAE (Ban Xia) inhibits TNF-α-induced NF-κB activation,^[14]^ and the extraction of PORIA (Fu Ling) has immunomodulatory effect.^[14]^ Also, SZYQD source “Han Doctor tong”, by SEMEN RAPHANI SEMEN SINAPIS FRUCTUS PERILLAE. A study suggested that the small dose of SZYQD had obvious expectorant effect.^[15]^

As far as we know, it is unclear whether ERD combined with SZYQD is effective and safe intervention for COPD. Therefore, we aim at providing evidence to clinicians so that more patients with COPD may benefit from alternative interventions.

## Author contributions

**Conceptualization**: Lu Deng, Xinyue Zhang, Larry E. Mille.

**Data curation**: Lu Deng, Xinyue Zhang, Larry E. Mille.

**Formal analysis**: Hui Tang, Qunfeng Shi, Wenhao Liao, Larry E. Mille.

**Investigation**: Meiling Zheng, Zidanqing Yang, Larry E. Mille.

**Methodology**: Lu Deng, Lizhen Wang, Larry E. Mille.

**Project administration:** Larry E. Mille.

**Resources:** Larry E. Mille.

**Software**: Meiling Zheng, Zidanqing Yang.

**Supervision**: Yan Dong.

**Visualization:** Larry E. Mille.

**Writing – original draft**: Lu Deng, Xinyue Zhang, Larry E. Mille.

**Writing – review & editing**: Hui Tang, Qunfeng Shi, Wenhao Liao, Keling Chen, Yan Dong.
